# *Actinobacillus seminis* DnaK interacts with bovine transferrin, lactoferrin, and hemoglobin as a putative iron acquisition mechanism

**DOI:** 10.1007/s12223-025-01271-7

**Published:** 2025-05-10

**Authors:** Candelario Vazquez-Cruz, Edmundo Reyes-Malpica, J. Fernando Montes-García, Pamela Bautista-Betancourt, Elena Cobos-Justo, Miguel A. Avalos-Rangel, Erasmo Negrete-Abascal

**Affiliations:** 1 Centro de Investigaciones en Ciencias Microbiológicas, BUAP, Apdo. Postal1622, Puebla, México; 2 Carrera de Biología, Facultad de Estudios Superiores Iztacala, UNAM, Av. de los Barrios # 1, Los Reyes Iztacala, 54090 Tlalnepantla, Estado de México Mexico

**Keywords:** *A. seminis*, Lactoferrin, Transferrin, Hemoglobin, Siderophore, DnaK

## Abstract

Ovine epididymitis, caused by *Actinobacilus seminis*, is an infectious disease that produces atrophy of the testis, low fertility, and sterility in infected animals. Iron is a microelement necessary for different vital functions in all organisms and most microorganisms. *A. seminis* iron acquisition mechanisms are undiscovered. For this reason, this work aimed to know the mechanisms this bacterium possesses to respond when grown in an iron restriction culture medium. *A. seminis* up-expressed three proteins, including a transferrin binding protein, and down-expressed seven (enzymes and putative adhesins) proteins when grown with 2,2′dipyridyl. With chelate, its growth was reduced by 40%, but it was recovered by adding 50-µM FeCl_3_. No siderophore production was detected with the CAS-BHI medium assay, but siderophore transporter proteins are present. Under normal growth conditions, this microorganism expresses a protein of 70 kDa, identified by mass spectrometry as DnaK. *A. seminis* DnaK interacts with biotin-labeled transferrin, lactoferrin, or bovine hemoglobin but not with biotin-labeled apo-transferrin or apo-lactoferrin, suggesting its participation in iron acquisition. This protein diminished its expression in iron restriction conditions at 37 °C but remained unchanged at 40 °C, and it is immune recognized by sheep serum with epididymitis. These different iron acquisition mechanisms could give rise to *A. seminis*, infecting different host tissues.

## Introduction

Epididymitis in small ruminants has been considered the leading cause of subfertility; this illness has been associated with different microorganisms directly affecting the epididymis or as a sequela of other diseases, such as orchitis. It has been suggested that these diseases are produced by three different entities, depending on the reproductive age of the affected animal (Gouletsou & Fthenakis 2015). *Brucella ovis*, *Histophilus somni*, and *Actinobacillus seminis* have been considered the primary organisms involved in this kind of disease. *H. somni* and *A. seminis* are considered opportunistic pathogens because both are part of the microbiome in the prepuce; however, for both microorganisms, their pathogenic potential as primary pathogens by solo inoculation in susceptible hosts has been demonstrated (Moustacas et al. 2014). *A. seminis* possesses different putative virulence factors according to its genomic information (Negrete-Abascal et al. 2018); however, few components have been identified (Healey et al. 1991; Schaller et al. 2000; Montes-García et al. 2018, 2019; García et al. 2020; De la Cruz Montoya et al. 2023; Ramírez-Paz-Y-Puente et al. 2023).

Metals are essential for all life forms because they play structural and catalytic roles in biological processes, including precursor biosynthesis, DNA replication, transcription, respiration, and responses to oxidative stress (Palmer & Skaar 2016; Hassan et al. 2020).

Iron is essential for the survival and reproduction of protozoa, fungi, and bacteria, except for *Lactobacilli* and *Borrelia burgdorferi* (Weinberg 2009; Troxell et al. 2012). Different studies with pathogen microorganisms have established that the ability to acquire iron from their host is considered a virulence factor (Weinberg 2009; Cook-Libin et al. 2022). Bacteria can get this microelement from their host or environment through three different mechanisms: import mechanisms, siderophores, or host proteins (Morin et al. 2021). In *A. seminis*, the response to growth in iron-restricted conditions is unknown. Hence, it became the objective of this work. This work tried to understand the mechanisms that *A. seminis* expresses to obtain a vital micronutrient and accomplish the physiological functions in which this element participates through in silico analysis and growth in iron-restricted conditions. Knowledge of the iron acquisition mechanisms by *A. seminis* led us to understand its capacity to infect different host tissues.

## Materials and methods

### Strain and culture conditions

*A. seminis* ATCC 15768 strain was grown in tryptic soy (TS) agar supplemented with 5% sheep blood and incubated at 37 °C in a humidified candle jar. To eliminate the iron from the glassware, the material was soaked overnight in 0.5% EDTA and then washed with deionized water (Abascal et al. 2009); deionized water was used to prepare all solutions and culture media. To evaluate the effect of iron restriction conditions on protein expression, an overnight culture of *A. seminis* was diluted 1:100 in flasks containing fresh TS broth and incubated at 37 °C under shaking until the cultures reached 0.1 optical density units at 600 nm. Next, the specific iron-chelating agent 2,2′-dipyridyl (Sigma-Aldrich, St. Louis, MO, USA) was added at 0.25 mM (final concentration), keeping one flask as the control without addition.

### In silico analysis

To have an overall idea about the *A. seminis* genes involved in iron metabolism, GenBank information about *A semis*-genome-draft (NLFK01000000) was used to search for genes using the RAST (Rapid Annotations using Subsystems Technology) server (https://rast.nmpdr.org/). The 22 nucleotide contigs were translated in silico to proteins to know putative proteins; proteins’ identity was deduced by homology to the Seed database and compared with proteins deduced by the GenBank pipeline. All proteins were analyzed to determine all those with functions associated with iron metabolism in Gram-negative bacteria, such as Fe-binding, complexing, capture, transport, regulation, and usage of iron as a cofactor. Data were considered valid if BLASTP and BLASTX also yielded closely related information using amino acid and nucleotide sequences, respectively (https://www.uniprot.org; https://blast.ncbi.nlm.nih.gov/Blast.cgi). Different hypothetical proteins were included in the base of the BLASTP analysis if they showed a high amino acid identity and a small E value to iron-related proteins. The amino acid sequence deduced molecular weight was calculated using the Compute pI/Mw tool (https://web.expasy.org/compute_pi/), and the most probable isoelectric point was inferred.

### Siderophore production test

Siderophore production was evaluated on Chrome Azurol S (CAS)-BHI agar as described by Tabatabai et al. (2008) using *Pseudomonas aeruginosa* O1 (PAO1) and *Escherichia coli* DH5α (Tabatabai et al. 2008; Chart et al. 2000) as positive and negative siderophore producer strains, respectively.

### Interaction of *A. seminis* with molecules containing iron

To determine whether *A. seminis* can interact with bovine transferrin, lactoferrin, or hemoglobin, the BHI medium was inoculated with the *A. seminis* strain and incubated under agitation overnight, as described above, in the presence or absence of 2,2′-dipyridyl. Cultures were adjusted to a 0.5 OD at 600 nm, and 5 µl samples were absorbed into a nitrocellulose membrane and blocked with 5% skim milk. Membranes were incubated with bovine transferrin, lactoferrin (iron saturated), or hemoglobin (Sigma-Aldrich); apo-lactoferrin or apo-transferrin biotin-labeled (Kittigul et al. 1998; Ramírez-Rico et al. 2024), solutions containing 80 µg/mL (Tremblay et al. 2006). The ability of *A. seminis* to bind these molecules was determined with peroxidase-labeled avidin (1:1000).

### Outer membrane proteins (OMPs) obtaining

Bacteria cultured overnight were used to inoculate flasks containing TS broth (1% v/v) in the presence or absence of 2,2′-dipyridyl and incubated under agitation for 24 h at 37 °C. The culture was centrifuged at 12,500 g for 30 min at 8 °C, and the cell pellet was suspended in 20 mM HEPES, pH 7.4, with 1-mg lysozyme. This bacterial suspension was incubated at 37 °C for one hour and then sonicated (15 s on/10 s off) on ice for 5 min. Unbroken cells were removed by centrifugation at 15,800 g for 2 min. The OMPs were obtained according to Blackall et al., (1990). Finally, the pellet was suspended in 500 μL of 20 mM HEPES pH 7.4 and frozen until use. Protein concentration was determined using the Bradford method, using bovine serum albumin as a standard.

### Sodium dodecyl sulfate–polyacrylamide gel electrophoresis

To visualize proteins expressed by bacteria cultured in iron-restricted or in a standard medium, samples (10 μg loaded per well) were separated by 10% sodium dodecyl sulfate–polyacrylamide gel electrophoresis (SDS-PAGE). After electrophoresis, gels were stained with Coomassie blue R.

### *A. seminis *growth in the presence of FeCl_3_

The *A. seminis* strain was grown in iron-restricted conditions as described above. To determine whether FeCl_3_ could help to recover the growth of *A. seminis* in 2,2′-dipyridyl-added medium, it was supplemented with FeCl_3_ (10, 20, 30, 40, 50, or 100 µM/mL). Cultures were incubated overnight at 37 °C under agitation, and the OD 600 nm was measured.

### Identity of proteins that change by iron restriction conditions

The peptide mass fingerprints of the selected OMPs were determined by matrix-assisted laser desorption ionization-time of flight mass spectrometry (MALDI-TOF MS, Thermo Fisher Scientific) after trypsin digestion of the proteins identified through SDS-PAGE (Montes-García et al. 2018; Ramírez-Paz-y-Puente et al. 2023). A protein identified by MALDI-TOF MS (DnaK) was modeled to know the possibility of interacting with iron-associated molecules. The 3D *A. seminis* DnaK model was generated using Phyre2 (Kelley et al. 2015); it presented high similarity with *E. coli* DnaK (access number PDBe 2kho). The spatial arrangement was verified with Prochek, Verify3D, and VADAR software (Lüthy et al. 1992; Laskowski et al. 1993; Willard et al. 2003). Ligand 3D models were obtained from a protein data bank: hemoglobin (access number PDB 3BJ1), transferrin (access number PDB 1SUV), and lactoferrin (access number PBD 1B1X). Docking analysis among *A. seminis* DnaK and ligands was performed using the HDOCK server site (http://hdock.phys.hust.edu.cn/). Higher-score docking models were visualized with PyMOL (https://pymol.org/2/).

### Far-western blot and immune recognition

To evaluate the immunogenicity of the *A. seminis* proteins over or under-expressed in the iron-restricted medium, proteins were separated by 10% SDS-PAGE and transferred to a nitrocellulose membrane (Abascal et al. 2009). Membranes were incubated with 1:1000 diluted polyclonal sera pooled from sheep with epididymitis (Montes-García et al. 2018), rabbit sera anti-transferrin binding proteins from *Avibacterium paragallinarum* (Abascal et al. 2009), or biotin-labeled bovine transferrin, lactoferrin, or hemoglobin (Sigma-Aldrich) (Kittigul et al. 1998). The immune reaction and interaction among proteins were revealed with rabbit IgG anti-sheep antibody, goat IgG anti-rabbit antibody, or avidin, all labeled with peroxidase, using diaminobenzidine and H_2_O_2_ as substrate.

## Results

### Ferric uptake regulator gene presence

The presence of a *fur* gene in the *A. seminis* ATCC 15768 genome (NLFK01000000.1) and the protein ferric uptake regulator (Fur) from this bacterium are recorded in the proteins database (WP_094945575.1).

### *A. seminis* genes related to iron metabolism

A detailed search of the genome (Accession number NLFK01000000)-encoded proteins related to iron metabolism revealed the presence of 74 proteins associated with this function, including binding (9), complexing (7), capture, transport (10), or use of iron as a cofactor, for example, cytochrome-related proteins. The in silico mass calculated for these proteins ranges from 7.9 to 105.8 kDa with a theoretical PI from 4.1 to 10.1 (https://web.expasy.org/compute_pi/). The list includes 17 hypothetical proteins related to iron metabolism; however, the annotation is uncertain (Table 1); most of them seem to be molecules associated with the membrane.

**Table 1:** Putative *A. seminis* proteins related to iron metabolism identified by in silico analysis

Protein ID ⍦	Name (product)	PI*	Mass (kDa)*
OZN24044.1	(2 Fe-2S)-binding protein	6.88	9880.5
OZN25744.1	ABC transporter	8.76	38,913.06
OZN25551.1	ABC transporter ATP-binding protein	5.59	39,023.66
OZN25551.1	ABC transporter ATP-binding protein	5.59	39,023.66
OZN24810.1	c-type cytochrome biogenesis protein CcmI	6.04	35,432.69
OZN24814.1	cytochrome c biogenesis protein CcmE	5.93	19,556.36
OZN24811.1	cytochrome c-type biogenesis protein CcmH	6.57	17,545.25
OZN24812.1	DsbE family thiol:disulfide interchange protein	8.4	20,526.7
OZN24794.1	DUF454 domain-containing protein	10.08	13,677.7
OZN25456.1	Fe-S biogenesis protein NfuA	4.6	21,222.89
OZN25993.1	Fe-S cluster assembly scaffold IscU	5.2	13,617.51
OZN25995.1	Fe-S cluster assembly transcriptional regulator IscR	8.48	17,432.91
OZN24804.1	Fe-S-binding ATPase	6.36	40,399.62
OZN25519.1	ferredoxin	4.48	9789.21
OZN24234.1	ferredoxin-type protein NapG	8.31	27,169.77
OZN25735.1	ferritin	4.66	19,459.98
OZN25736.1	ferritin	5.11	18,516.1
OZN24040.1	ferrochelatase	6.6	37,541.17
OZN24035.1	filamentous hemagglutinin	6.27	47,811.33
OZN24063.1	glutamate-1-semialdehyde-2,1-aminomutase	6.07	45,802.84
OZN24033.1	hemagglutinin	9.04	13,854.97
OZN25059.1	heme ABC transporter	8.7	27,719.48
OZN24789.1	heme ABC transporter ATP-binding protein	8.5	30,412.12
OZN24816.1	heme ABC transporter permease	6.44	19,991.68
OZN24246.1	heme biosynthesis protein HemY	7.7	48,572.67
OZN24815.1	heme exporter protein CcmD	9.8	7910.21
OZN24788.1	heme iron utilization protein	9.19	18,774.5
OZN24813.1	heme lyase NrfEFG subunit NrfE	9.59	73,349.22
OZN25071.1	heme lyase NrfEFG subunit NrfE	9.43	72,097.36
OZN25073.1	heme lyase NrfEFG subunit NrfF	7.75	17,554.2
OZN25670.1	heme utilization protein	5.15	13,932.61
OZN24834.1	heme utilization protein HutZ	6.44	19,991.68
OZN24790.1	hemin ABC transporter substrate-binding protein	8.61	30,134.24
OZN24283.1	HemX protein	4.59	49,538.08
OZN24031.1	hypothetical protein; Heme utilization or adhesion protein	6.12	49,687.63
OZN24331.1	hypothetical protein; Glycine cleavage T-protein	5.66	30,701.44
OZN24364.1	hypothetical protein; Filamentous haemagglutinin family outer membrane protein	6.7	72,357.17
OZN24443.1	hypothetical protein; Uncharacterized protein	4.74	12,723.64
OZN24786.1	hypothetical protein; TonB-dependent hemoglobin/transferrin/lactoferrin family receptor	9.47	30,376.04
OZN24787.1	hypothetical protein; TonB-dependent hemoglobin/transferrin/lactoferrin family receptor	8.86	53,138.56
OZN24792.1	hypothetical protein; Mn2 +/Fe2 + NRAMP family transporter	9.32	45,558.29
OZN24796.1	hypothetical protein; Sulfite reductase [NADPH] hemoprotein beta-component	7.72	12,005.19
OZN25009.1	hypothetical protein; calcium binding hemolysin	4.96	12,068.65
OZN25047.1	hypothetical protein; Hemolysin transporter protein shlB	5.35	17,696.17
OZN25074.1	hypothetical protein; heme lyase subunit	7.81	31,689.48
OZN25239.1	hypothetical protein; Filamentous hemagglutinin	5.16	11,930.34
OZN25241.1	hypothetical protein; filamentous hemagglutinin	5.76	25,341.3
OZN25327.1	hypothetical protein; divalent metal cation transporter	9.54	41,138.25
OZN25525.1	hypothetical protein;Iron (Chelated) transport system membrane protein	6.27	30,615.76
OZN25535.1	hypothetical protein; Transferrin-binding protein	5.64	8646.76
OZN25550.1	hypothetical protein;Periplasmic ferric iron-binding protein	5.94	37,907.02
OZN24835.1	iron ABC transporter	9.48	35,707.93
OZN25745.1	iron ABC transporter permease	9.22	62,441.37
OZN26038.1	iron ABC transporter substrate-binding protein	8.49	37,932.34
OZN24533.1	iron donor protein CyaY	4.22	11,551.85
OZN25992.1	iron-sulfur cluster assembly protein IscA	4.84	11,649.2
OZN24080.1	iron-sulfur cluster insertion protein ErpA	4.11	12,061.45
OZN25544.1	iron-sulfur cluster-binding protein	8.39	52,154.2
OZN25989.1	ISC system 2 Fe-2S type ferredoxin	4.2	12,422.93
OZN25402.1	ligand-gated channel protein	9.13	95,573.34
OZN25758.1	osmotically-inducible protein OsmY	8.62	20,967.29
OZN24621.1	outer membrane colicin Js receptor	7.73	82,995.13
OZN24505.1	oxidative damage protection protein	5.91	10,897.64
OZN24573.1	protoporphyrinogen oxidase	9.12	19,569.64
OZN24233.1	quinol dehydrogenase ferredoxin subunit NapH	8.97	31,839.73
OZN25961.1	succinate dehydrogenase/fumarate reductase iron-sulfur subunit	8.41	28,706.19
OZN24503.1	TonB-dependent receptor	6.3	91,613.24
OZN24729.1	TonB-dependent receptor	8.67	10,2374.62
OZN24730.1	TonB-dependent receptor	6.89	91,833.18
OZN25306.1	TonB-dependent receptor	9.12	87,707.5
OZN25537.1	TonB-dependent receptor	8.93	10,5838
OZN25762.1	TonB-dependent receptor	9.31	83,841.68
OZN25423.1	TonB-dependent siderophore receptor	9.45	76,010.17
OZN25536.1	transferrin-binding protein	6.82	58,184.23

Also, the in silico analysis of the genomic *A. seminis* sequence (Table 1) indicates the presence of genes encoding for the putative TonB-dependent siderophore receptor (WP_143403349.1), an iron-chelating uptake ABC family transporter (WP_013745078.1), a ferrichrome outer membrane transporter (AEC17141.1) and a putative transferrin binding protein B (TbpB) of 58 kDa (OZN25536.1) but not a TbpA protein.

### Siderophores production

**Fig. 1:**
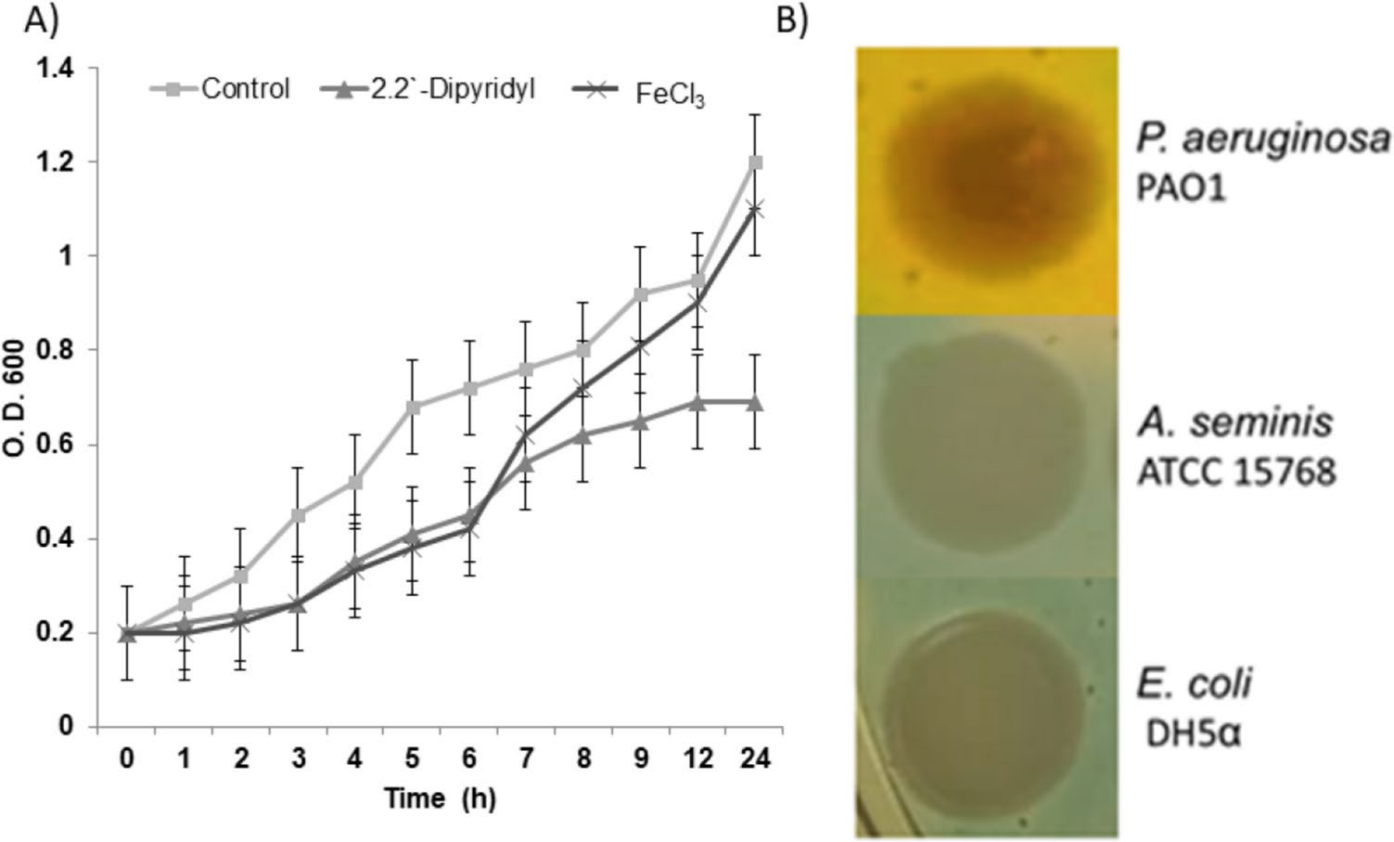
Effect of iron restriction in *A. seminis* growth. *A. seminis* was grown in the presence of 2,2′-dipyridyl, and 6 h after, 50-µM FeCl_3_ was added, final concentration, (**A**). Siderophore production test in Chrome Azurol S-BHI agar (**B**). *P. aeruginosa* PAO1 and *E. coli* DH5œ were used as positive and negative siderophore producers, respectively

The chrome azurol-BHI agar test was done to determine whether *A. seminis* could produce siderophores. The PAO1 strain induced a color change of blue to yellow in chrome azurol-BHI agar, indicative of siderophore production. Neither *E. coli* DH5α used as a negative siderophore producer nor *A. seminis* could change the medium color, suggesting that *A. seminis* cannot produce siderophores (Fig. 1).

### Interaction with iron-associated proteins

**Fig. 2:**
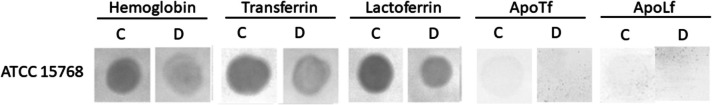
Dot blot assay. *A. seminis* ATCC 15768 strain was grown in the absence (C) or presence (D) of 2′2-dipyridyl and incubated in the presence of biotin-labeled bovine hemoglobin, transferrin, lactoferrin, apo-transferrin (Apo-Tf) or apo-lactoferrin (Apo-Lf)

Overnight growth of *A. seminis* in the presence of 2,2′-dipyridyl diminished 30 to 40% in the low iron concentration medium compared to growth in normal conditions (Fig. 1). The dot blot assay revealed that *A. seminis* was able to bind bovine hemoglobin, transferrin, and lactoferrin (Fig. 2) but not bovine apo-transferrin or apo-lactoferrin.

### Expression of iron restriction proteins and immune recognition

**Fig. 3:**
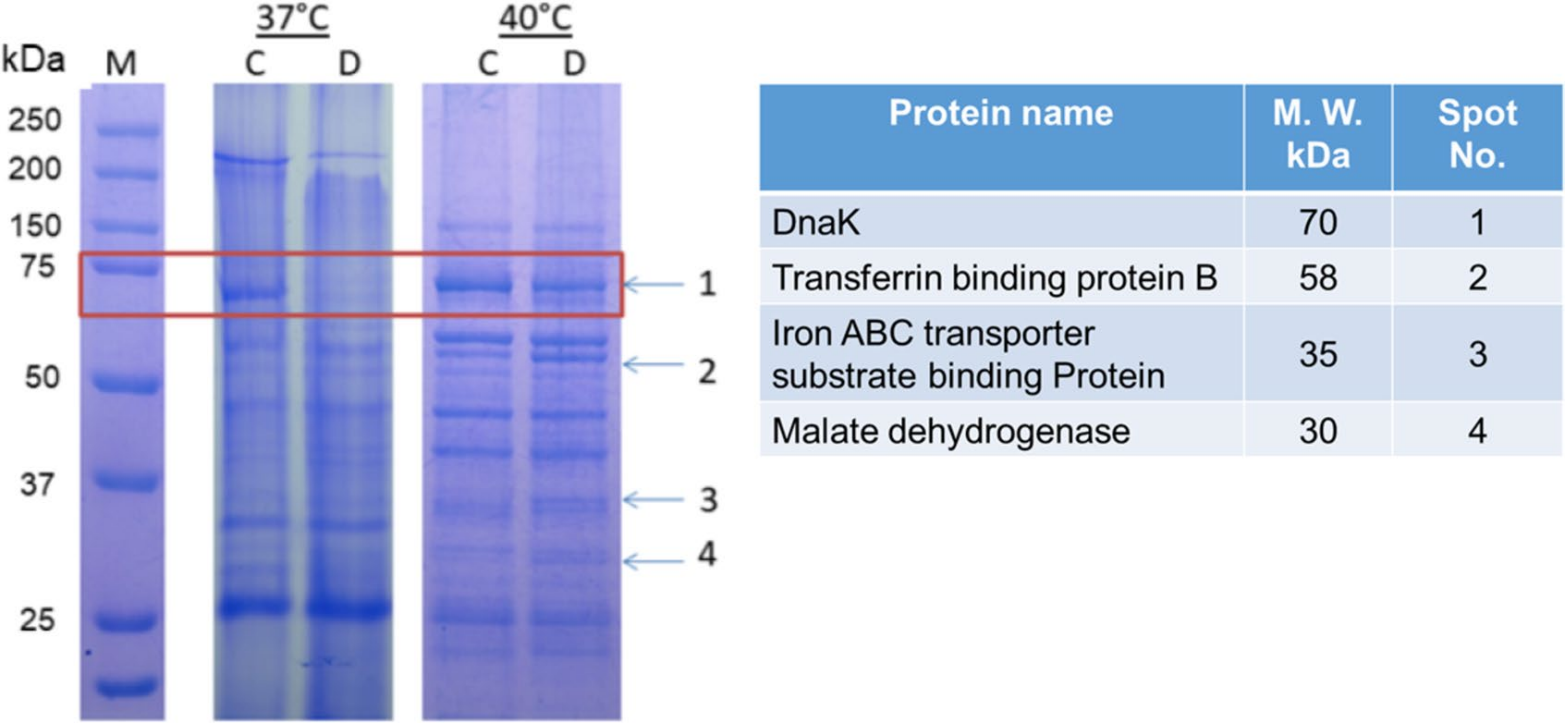
*A. seminis* ATCC 15768 strain OMPs patterns grown at 37 °C or 40 °C in the presence of 2,2′-dipyridyl (D) or its absence (C). The red rectangle shows the 70-kDa protein down-expressed in iron-restricted growth conditions. Identified proteins that presented differential expression due to the growth in the presence of 2,2′-dipyridyl are shown

**Fig. 4:**
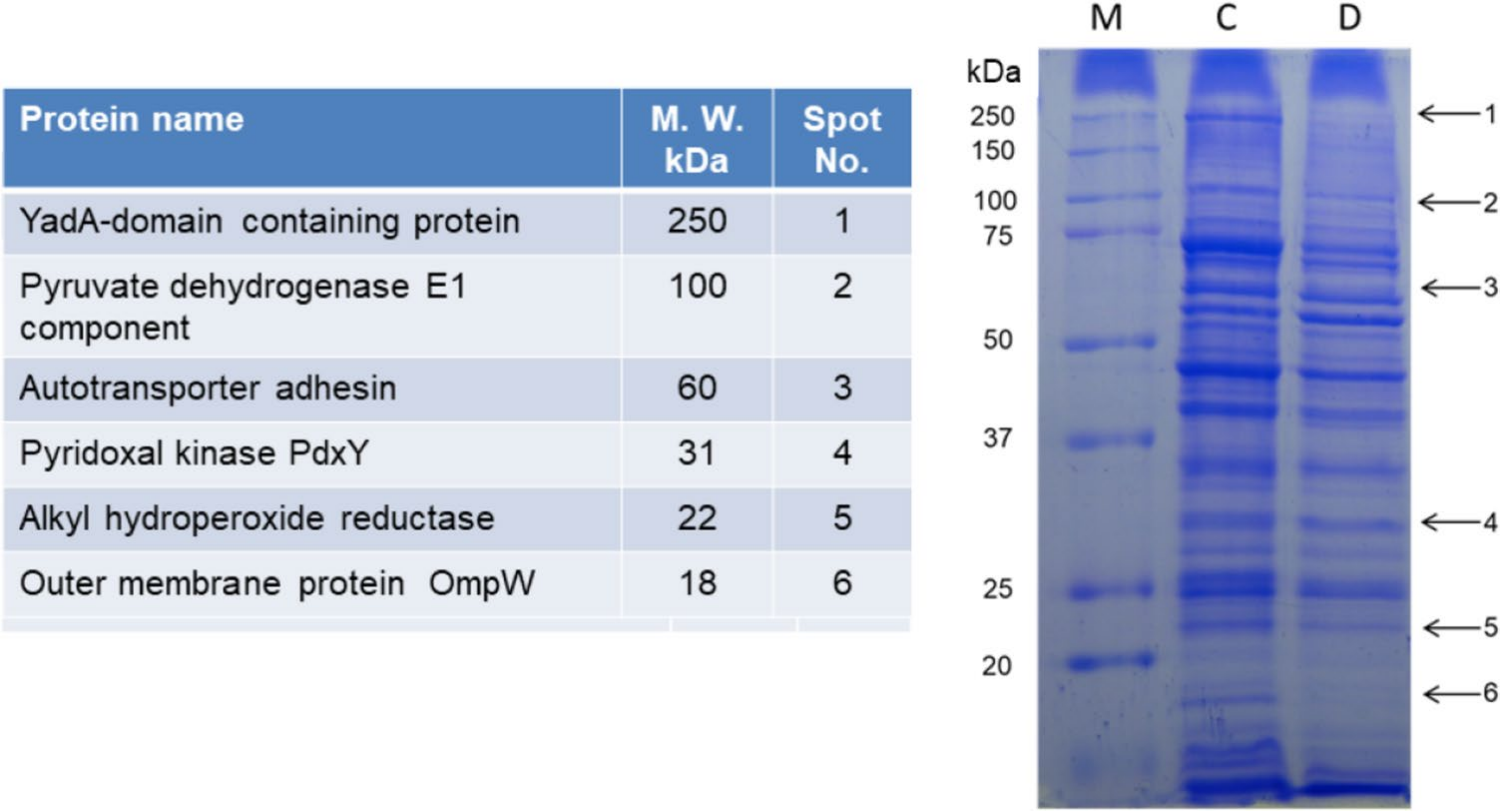
*A. seminis* ATCC 15768 strain total cell extract protein pattern has grown at 40 °C in the presence of 2,2′-dipyridyl (D) or its absence (C). Identified proteins that presented differential expression due to the growth in the presence of 2,2′-dipyridyl are shown

The protein pattern of *A. seminis* grown in iron-restricted conditions up-expressed three different proteins and down-expressed seven (Figs. 3 and 4). Several of these proteins were immune-recognized by a sheep serum pool with epididymitis, suggesting their expression in vivo and participation in *A. seminis* metabolism during infection. Bovine biotin-labeled lactoferrin, transferrin, or hemoglobin interact with a 70 kDa OMP. Still, not with apo lactoferrin or transferrin (Fig. 2). This protein was practically undetectable in *A. seminis* samples in iron restriction conditions at 37 °C. Still, it was present if the culture was incubated at 40 °C.

**Fig. 5:**
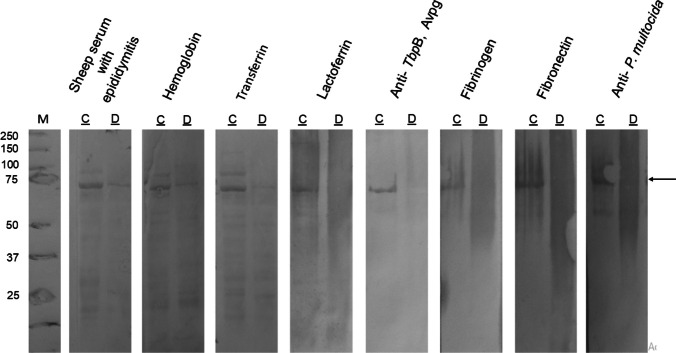
Interaction of 70-kDa protein from *A. seminis* grown at 37 °C or 40 °C with different molecules. (C) Control without 2,2′-dipyridyl addition; (D) in the presence of 2,2′-dipyridyl. The arrow indicates the 70-kDa protein in the red rectangle in Fig. 3

This *A. seminis* 70 kDa OMP was immune-recognized by sheep serum with epididymitis, indicating its in vivo expression and immunogenicity. Also, this protein immune cross-reacted with a polyclonal serum from a *Pasteurella multocida* rabbit infected, indicating a common antigen among different *Pasteurellaceae* members. Besides, the 70-kDa protein also interacted with biotin-labeled bovine fibrinogen and fibronectin, suggesting its participation as an adhesin (Fig. 5).

### Iron restriction-induced proteins’ identification

The 70-kDa protein’s mass spectrometric analysis identified it as *A. seminis* chaperone protein DnaK (WP_094945526.1).

**Fig. 6:**
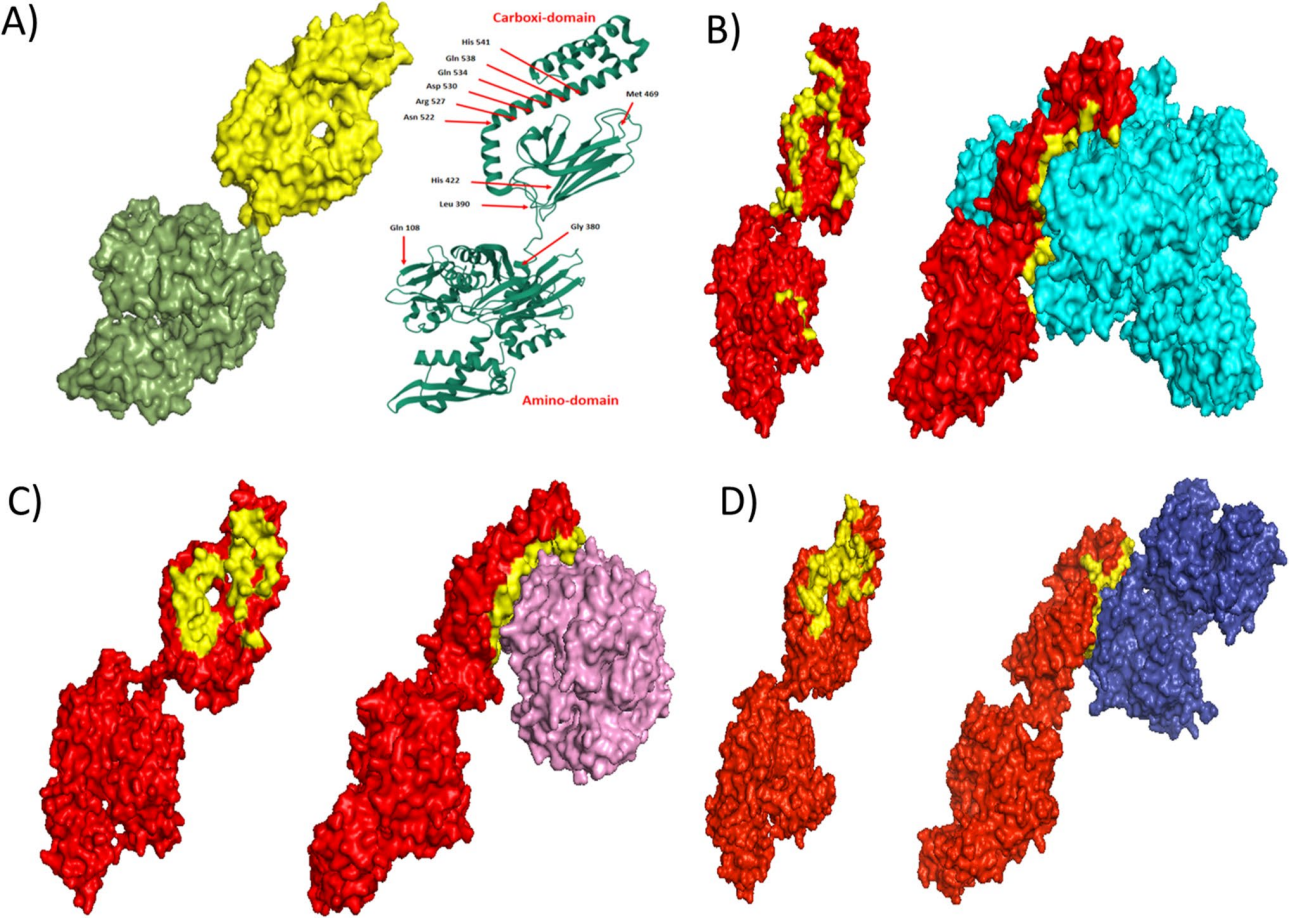
Docking model of *A. seminis* DnaK (**A**) and its interaction with hemoglobin (**B**, cyan), transferrin (**C**, pink), or lactoferrin (**D**, blue). Yellow indicates the end-carboxy regions involved in this interaction. Arrows indicated the amino acids participating in the interaction of *E. coli* DnaK with iron-associated proteins. Modeling was performed with Phyre2 and visualized with PyMol

For other *A. seminis* proteins that changed due to the presence of 2′2′dipyridyl in the culture medium, the mass spectrophotometric proteins analysis of 30, 35, and 53 kDa revealed identity with malate dehydrogenase (accession number WP_094945414.1), iron ABC transporter substrate-binding protein (WP_094945667.1), and transferrin binding protein B (WP_342352653.1), respectively (Fig. 3). Other proteins identified by growing in a reduced iron medium are listed in Fig. 4. The 3D model of *A. seminis* DnaK generated by phyre2 used 600 residues of the sequence (95% of the total sequence), presenting 100% confidence with the *E. coli* DnaK protein. *A. seminis* DnaK generated model shows binding sites (arrows) to iron-associated proteins (in yellow) like that of the *E. coli* DnaK protein in the carboxy domain (Fig. 6A, cartoon). *A. seminis* DnaK’s predicted model was used to evaluate its interaction with iron-binding proteins using the HDOCK server. The coupling models show a high coupling probability (> 95%) between *A. seminis* DnaK and hemoglobin (Fig. 6B), transferrin (Fig. 6C), and lactoferrin (Fig. 6D).

## Discussion

Iron is a microelement vital for all microorganisms (Weinberg 2009; Troxell et al. 2012). Due to the variability among Fe^+3^/Fe^+2^ forms, iron participates in various cellular metabolic processes when incorporated into proteins as a catalytic center or electron carrier. Due to its high tendency to generate hydroxyl radicals, the concentration of this micro-nutriment must be finely regulated. Most of the time, Fur protein controls this regulation. In silico analysis, it was found that *A. seminis* contains a *fur* gene as a putative iron metabolism control mechanism. This search also indicates the presence of putative siderophore receptors and transporters. However, no siderophores production was detected by the chromo azurol-BHI agar test. It has been described that *Pasteurellaceae* members, similarly to other mucosal pathogens, can obtain iron from the host’s iron-binding proteins, like transferrin or lactoferrin, without siderophore production (Cornelissen 2003; Ellermann & Arthur 2017) or use the siderophores produced by other microorganisms (Morton et al. 2010; Rhodes et al. 2007). Our result suggests that *A. seminis* cannot produce siderophores.

Most of the iron in the environment is insoluble and is associated with transporting or storing proteins in the host. Microorganisms express multiple systems to overcome their low iron availability and get this essential element from inorganic iron associated with heme or transferrin/lactoferrin proteins. Dot blot results suggest that *A. seminis* could use different iron sources. This interaction could be through DnaK. DnaK is a 70-kDa heat shock protein, a poorly induced protein at 37 °C in iron restriction conditions but expressed optimally at 39 °C. Ovine’s average temperature is 39–40 °C, and the presence of this protein, despite iron restriction conditions, indicates its relationship with a heat-shock protein. Epididymis temperature is usually 3 to 5° lower than corporal temperature. *Mycobacterium smegmatis* chaperons GroEL and DnaK proteins were not produced at 37 °C in iron restriction conditions when studying the response to oxidative stress (Lundrigan et al. 1997), similar to what was observed here.

Although DnaK proteins have not been associated with iron acquisition, the involvement of other chaperonins in iron-restricted growth or immune response has been described in different microorganisms. For example, secreted or cytoplasmic chaperonin GroEL from *Helicobacter pylori* binds iron from FeCl_3_, hemoglobin, or lactoferrin (González López et al. 2013); this protein was also described as a protein participating in the iron caption but not induced as a response of this restriction.

A *tbp*B gene in *A. seminis* genome but not a *tbp*A gene could suggest the incapability to interact with transferrin. However, in pathogenic *Neisseria*, it has been described that gonococcal TbpA mutants cannot take iron from transferrin, and TbpB mutants can get iron from transferrin but with low efficiency (Anderson et al. 1994). A docking prediction using the software Phyre2 between *A. seminis* DnaK and heme or transferrin/lactoferrin molecules strongly supports the possibility that *A. seminis* DnaK can interact with all those molecules, supporting the likelihood that DnaK participates in iron-obtaining mechanisms. *A. seminis* DnaK could substitute the absence of TbpA and get approach transferrin to TbpB so that TbpB could remove the iron attached to transferrin. Remotion or interruption of the *A. seminis* DnaK coding gene could help us to corroborate this assumption; unfortunately, there are no known conditions to transform *A. seminis* now*.*

The growth of *A. seminis* in the presence of 2,2′-dipyridyl was recovered 100% after adding 50 µM FeCl_3_. *Bradyrhizobium japonicum*, a symbiotic soybean microorganism unable to produce siderophores, uses *feo*AB operon genes to acquire iron from FeCl_3_ aerobic environments (Sankari and O´Brian 2016). In *A. seminis*, proteins participating in these functions are unknown; *feo* genes are not present in its genome, and only one fumarate reductase was identified by in silico analysis.

The absence of 250 kDa, 60 kDa, and 18 kDa proteins or diminished concentrations (100, 31, and 22 kDa proteins) in *A. seminis* samples grown in iron restriction conditions was observed. *Staphylococcus epidermidis* grown in iron restriction conditions releases to the supernatant a 32 kDa lipoprotein associated with the membrane. The gene encoding this protein belongs to an iron-regulated operon and shows homology to different bacterial adhesins (Cockayne et al. 1998). Other enzymes have also been described as proteins participating in bacterial adherence (Montes-García et al. 2018; Słotwińska 2013; Jeffery 2018); some are affected by iron restriction (Zhang et al. 2020). In *A. seminis*, something similar could be occurring.

DnaK protein has been described as a moonlighting protein, and it could be significant in *A. seminis* pathogenesis and act as a good immunogen. This protein participates in bacterial pathogenesis, immune response, and apoptosis. Among pathogenesis strategies, DnaK participates in cell invasion and adhesion, antibiotic resistance, immune system evasion, cell death responses, and oncogenic properties (Ghazaei 2017; Zella et al. 2018). Recombinant DnaK mice immunization induces protection against *B. melitensis* (Ghasemi et al. 2014). We found that *A. seminis* DnaK interacts with bovine fibronectin and fibrinogen, suggesting its participation as an adhesin; fibrinogen is a soluble extracellular matrix component. Still, it is also essential in coagulation, fibrosis, and protection against infection and inflammation (Davalos & Akassoglou 2012). *A. seminis* secretes a metalloprotease that degrades fibrinogen, which could contribute to host damage (De la Cruz Montoya et al. 2023).

By far-western blot, interaction among 70 kDa protein and biotin-labeled hemoglobin was observed; in silico analysis identified several genes that could participate in hemoglobin procurement; however, the mass spectrometric assay of up or down-expressed proteins by iron restriction, identified a 35 kDa iron ABC-transported substrate-binding protein and not a hemoglobin receptor. This bacterium, grown in the presence of testosterone, induces the expression of a protease degrading bovine hemoglobin (Data unpublished) that could be an alternative mechanism. Although *A. seminis* possesses different genes encoding specific proteins (hemoglobin, lactoferrin- or transferrin-binding proteins, etc.), the results obtained herein suggest that instead of expressing specific proteins, this bacterium prefers to economize and use proteins of everyday use and response, such as the DnaK chaperone and the GroEL protein, another chaperonin that previously had been described as a hemagglutinin for this microorganism (Montes-García et al. 2019), enzymes that participate as adhesins (Montes-García et al. 2018) or EF-Tu used by extraintestinal *E. coli* to obtain iron from holo-transferrin (Sun et al. 2022); all of them are moonlighting proteins. *A. seminis* seems to count upon different iron-uptake mechanisms to be considered a successful pathogen.

## Conclusion

This study concludes that *A. seminis* DnaK could participate in iron acquisition in the epididymis through interaction with proteins associated with iron (bovine transferrin, lactoferrin, or hemoglobin) but not without it. The presence of siderophores receptors and transporters but the absence of siderophores production suggests the capability to use siderophores produced by other microorganisms, in the base that this microorganism does not synthesize them. The results indicate this microorganism counts with different iron acquisition mechanisms, but more studies are necessary to confirm and understand those processes.
